# Haploidentical CD19/CD22 bispecific CAR-T cells induced MRD-negative remission in a patient with relapsed and refractory adult B-ALL after haploidentical hematopoietic stem cell transplantation

**DOI:** 10.1186/s13045-019-0741-6

**Published:** 2019-06-10

**Authors:** Hejin Jia, Zhenguang Wang, Yao Wang, Yang Liu, Hanren Dai, Chuan Tong, Yelei Guo, Bo Guo, Dongdong Ti, Xiao Han, Qingming Yang, Zhiqiang Wu, Weidong Han

**Affiliations:** 1 Molecular & Immunological Department, Bio-therapeutic Department, Chinese PLA General Hospital, No. 28 Fuxing Road, Beijing, 100853 China; 2 Department of Geriatric Hematology, Chinese PLA General Hospital, No. 28 Fuxing Road, Beijing, 100853 China

**Keywords:** Chimeric antigen receptor, CAR-T, Bispecific CAR-T, GVHD, Haploidentical CAR-T

## Abstract

**Background:**

Chimeric antigen receptor T (CAR-T) cell therapy simultaneously against CD19 and CD22 is an attractive strategy to address the antigen escape relapse after CD19-directed CAR-T cell therapies. However, the potential of optimizing the durability of remission by this approach in patients with B cell acute lymphoblastic leukemia (B-ALL) remains a critical unanswered question so far.

**Case presentation:**

We treated an adult patient with relapsed and refractory B-ALL after haploidentical hematopoietic stem cell transplantation (HSCT) by administering haploidentical CAR-T cells targeting both CD19 and CD22 following preparative lymphodepleting chemotherapy. This patient has remained in minimal residual disease-negative remission for more than 14 months and has been tapered off graft versus host disease prophylaxis.

**Conclusions:**

CAR simultaneously targeting CD19 and CD22 has the potential of inducing long-term remission in patients with B-ALL.

**Electronic supplementary material:**

The online version of this article (10.1186/s13045-019-0741-6) contains supplementary material, which is available to authorized users.

## Background

CD19-directed chimeric antigen receptor T (CAR-T) cells have shown unprecedented initial response rates in relapsed/refractory (R/R) B cell acute lymphoblastic leukemia (B-ALL); however, relapse due to the loss or downregulation of the CD19 is an emerging threat to this innovative form of cellular immunotherapy [1, 2]. CAR-T cells specific for CD22, another B cell lineage of antigen, have also shown comparable potency to CD19-directed CAR-T cells in 21 adult patients with B-ALL [3]. CAR-T cells simultaneously targeting CD19 and CD22 have demonstrated potential benefit of overcoming CD19 immune escape [3], and early clinical experience with this approach in pediatric and adult B cell malignancies has shown promising results [4–7], but the effect of this approach on long-term disease control either in the autologous or in the allogeneic setting remains a critical unanswered question so far.

Currently, CD19-directed CAR-T cells are mainly manufactured from patient-derived T cells. However, in some circumstances such as failure of autologous CAR-T cell manufacturing or without time window for leukapheresis because of the active disease, CAR-T cells are also generated from donor-derived T cells [8–11]. Cumulative data from the clinical trials of donor-derived CAR-T cells have shown that donor-derived CAR-T cells targeting CD19 could effectively salvage relapsed B-ALL after allogeneic hematopoietic stem cell transplantation (HSCT) with a lesser risk of graft versus host disease (GVHD) flare [11–13].

We have designed a bispecific CAR simultaneously targeting both CD19 and CD22 (TanCAR-19/22) and initiated a clinical trial exploring T cells expressing this CAR (TanCAR-T 19/22 cells) in R/R B cell malignancies. Here, we report on the immunologic and long-term clinical effects of this haploidentical (haplo) TanCAR-T 19/22 cells used in a compassionate use setting in a patient with relapsed and refractory adult B-ALL after haplo-HSCT. As of 28 March 2019, the patient has remained in minimal residual disease (MRD)-negative remission for more than 14 months.

## Case presentation

This subject was a 22-year-old man with B-ALL who had third bone marrow (BM) relapse before enrollment on to our compassionate clinical protocol using TanCAR-T 19/22 cells. He was diagnosed with B-ALL with more than 100 × 10^9^/L WBC count and normal karyotype in January 2016. After complete remission (CR) 2, he underwent haplo-HSCT from his father 10 months after the original diagnosis. He had suffered hemorrhagic cystitis and stage 1 gastrointestinal acute GVHD within 2 months post haplo-HSCT, which resolved with 15 daily doses of methylprednisolone 50 mg followed by 5 daily doses of methylprednisolone 100 mg. Three months after discontinuation of the cyclosporine A and methylprednisolone, his disease relapsed with 6.4% marrow blasts when he still had full donor chimerism, then rapidly progressed with 56.5% marrow blasts by flow cytometry 10.6 months post haplo-HSCT, and undetectable donor chimerism was noted at the same time. He received salvage chemotherapy with MOEP (3 daily doses of mitoxantrone 10 mg, vindesine 4 mg, 3 daily doses of etoposide 100 mg, and 5 daily doses of dexamethasone 15 mg) and had severe bone marrow depression and no response with 65.4% marrow blasts 1 month after the first cycle of MOEP. Then, he was treated on our haplo-CAR-T 19 cell protocol. He received cytoreduction chemotherapy with vindesine and methylprednisolone plus hydroxyurea and lymphodepleting therapy with daunorubicin and cyclophosphamide, and his marrow blasts dropped to 12.7% prior to haplo-CAR-T 19 cell infusion. Haplo-CAR-T 19 cells at a dose of 4.91 × 10^6^/kg (2.89 × 10^7^ T cells/kg, 17% transfection efficiency) were administered and induced MRD-negative CR (MRD-CR) and full donor chimerism within 2 weeks after infusion. The infused haplo-CAR-T 19 cells exhibited rapid expansion and peaked with 15,281 copies per microgram DNA within the first 2 days after infusion, but dropped from 3374 copies per microgram DNA at day 7 to 468 copies per microgram DNA at day 12; methylprednisolone 160 mg and dexamethasone 5 mg were used at day 11 for treatment of the infusion-related grade 3 cytokine release syndrome (CRS). He experienced stage 3 skin acute GVHD within 1 month after haplo-CAR-T 19 cell infusion, which was under control with 5 daily doses of methylprednisolone 40 mg plus cyclosporin A 80 mg administered from day 31 after haplo-CAR-T 19 cell infusion. However, 1 month after obtaining MRD-CR, his disease exhibited florid progression with WBC count increasing from 1.59 × 10^9^ to 12.52 × 10^9^/L and corresponding percentage of circulating blasts increasing from 1.39 to 67.37% within 2 weeks; his bone marrow exhibited highly active cellular proliferation with 59.67% blasts that had the expression pattern CD19+ CD34+ CD10+ CD22+ CD38+ CD58+ CD33+ CD20− CD13− CD15−. At the same time, undetectable haplo-CAR-T 19 cells and donor chimerism were documented.

**Fig. 1:**
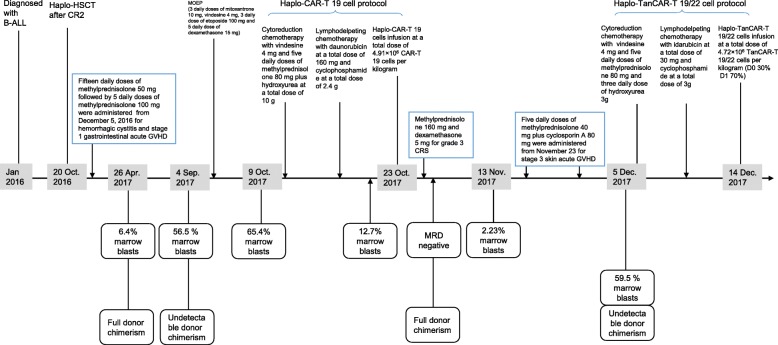
Diagrammatic sketch of the treatments

**Fig. 2:**
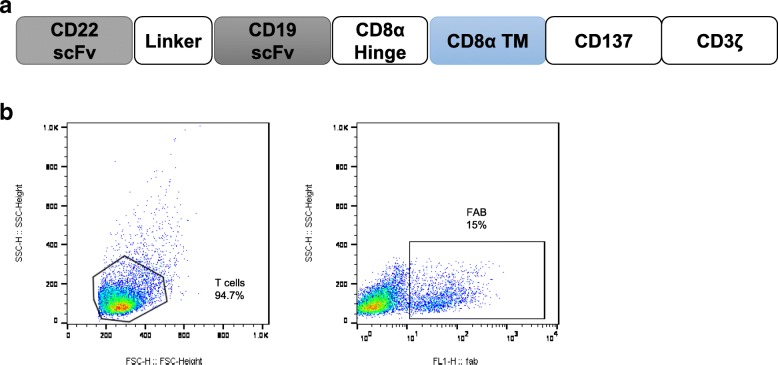
Expression of the TanCAR-19/22. **a** Schematic of the TanCAR-19/22. **b** TanCAR-19/22 gene expression by FACS. As described in the “Detection of haplo-TanCAR-T 19/22 cells” section, Biotin-SP-AffiniPure Goat Anti-Mouse IgG, F (ab') 2 Fragment Specific and PE Streptavidin antibody were used

In this case, other therapies including TanCAR-T 19/22 cells rather than salvage chemotherapy or reinfusion of CAR-T 19 cells could be a potential treatment option for this patient due to the poor response to salvage chemotherapy and poor persistence of infused CAR-T 19 cells. However, higher tumor burden and short-term interval post discontinuation of steroid greatly increased the risk of failure of the generation of autologous CAR-T cells; florid progression of the disease made waiting till steroid tapered off was less feasible. Donor-derived TanCAR-T 19/22 cell therapy was  an optimal approach to overcome this problem, but as well known, haplo-CAR-T cell therapies were not to be advocated routinely in the setting of prior GVHD requiring steroid mainly due to raised concern for the high risk of GVHD reactivation. After more careful consideration of the clinical benefits and risks of the second haplo-CAR-T cell infusion, he was enrolled on to our compassionate clinical protocol using haplo-TanCAR-T 19/22 cells. His father underwent apheresis, and the peripheral blood mononuclear cells (PBMCs) were used to prepare the TanCAR-T 19/22 cells. He received cytoreduction chemotherapy with vindesine 4 mg and five daily doses of methylprednisolone 80 mg and three daily doses of hydroxyurea 3 g followed by a lymphodelpeting chemotherapy with idarubicin at a total dose of 30 mg and cyclophosphamide at a total dose of 3 g. Planned bone marrow aspiration after the abovementioned chemotherapy and prior to haplo-TanCAR-T 19/22 cell infusion was not performed due to poor compliance of the patient. Two days later, he was treated with haplo-TanCAR-T 19/22 cells at a total dose of 4.72 × 10^6^ TanCAR-T 19/22 cells per kilogram (3.05 × 10^7^ T cells per kilogram, 15% transfection efficiency) administered via fractioned dosing (D0, 30%; D1, 70%) for safety consideration (Figs. 1 and 2).

### Generation of haplo-TanCAR-T 19/22 cells

The materials and methods used in TanCAR-T 19/22 production have been described previously [14–17], with the exception of the construct of the CAR and the source of PBMCs used for manufacturing the TanCAR-T 19/22 cells. TanCAR-19/22 was a tandem CAR molecule, consisting of an anti-CD22 scFv derived from mouse m971 mAb [18] and anti-CD19 scFv derived from the mouse FMC63 mAb [19], joined in tandem, human CD8α hinge and transmembrane domain, and human CD137 and CD3ζ signaling domains. A schematic of the TanCAR-19/22 is shown in Fig. 2a. PBMCs used for manufacturing the TanCAR-T 19/22 cells were collected by leukapheresis rather than fresh peripheral blood (PB).

### Detection of haplo-TanCAR-T 19/22 cells

Flow cytometry was used for the determination of the TanCAR-19/22 transfection efficiency and quantification of the haplo-TanCAR-T 19/22 cells in clinical specimens using a Biotin-SP-AffiniPure Goat Anti-Mouse IgG, F (ab') _2_ Fragment Specific (Jackson ImmunoResearch, USA) and PE Streptavidin antibody (BD Biosciences, USA). Haplo-TanCAR-T 19/22 cells in clinical specimens also were measured by qPCR as described [8].

### Assessment of chimerism status

The extent of donor engraftment in clinical specimens was assessed by using short tandem repeat amplification and fluorescence labeling multiplex PCR combined with capillary electrophoresis as described [20].

### Cytokine measurements

Serum interleukin (IL)-2, IL-6, IL-8, and IL-10 and tumor necrosis factor-α levels were batch analyzed as described [14].

### Haplo-TanCAR-T 19/22 cells induced durable MRD-negative remission with full donor chimerism

**Fig. 3:**
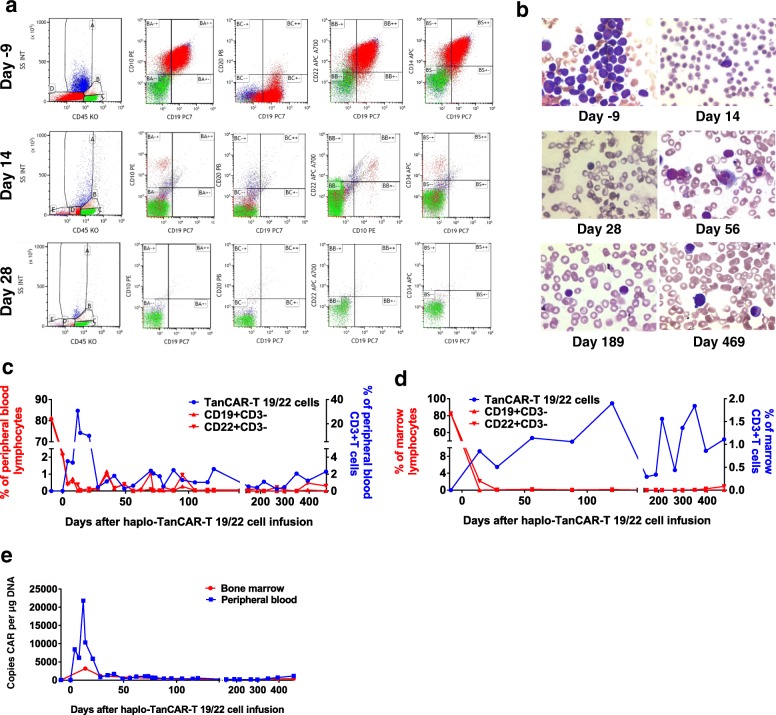
Clinical activity and expansion of haplo-TanCAR-T 19/22 cells. **a** There were 0.73% residual marrow blasts with expression of CD22 but loss of CD19 expression at day 14 after infusion, which were undetected by day 28. The cells in the D gate represent the blast population count of the total nucleated cells in BM aspirates. Day 0 is the day of haplo-TanCAR-T 19/22 cell infusion. **b** BM aspirates before and after haplo-TanCAR-T 19/22 cell infusion. Within the first 28 days after infusion, and at serial time points thereafter for 14 months, there was no evidence of blasts in BM. **c**, **d** Haplo-TanCAR-T 19/22 cells expanded within the first 12 days and continue to be detectable by flow cytometry with low levels in PB and BM through more than 14 months. B cells had not recovered as the most recent follow-up. **e** The presence of haplo-TanCAR-T 19/22 cells in PB and BM as assessed by qPCR

BM before haplo-TanCAR-T 19/22 cell protocol showed predominant blast cells with an absence of normal BM precursors. BM flow cytometry at day 14 after haplo-TanCAR-T 19/22 cell infusion indicated that there were 0.73% residual marrow blasts. Of note, those residual leukemic blasts exhibited the expression pattern CD34+ CD10+ CD22+ CD38+ CD33+ CD19− CD20−, which were undetected by flow cytometry by day 28 in the absence of further therapy (Fig. 3a). Given the incomplete recovery of platelet and absolute neutrophil count by day 28, this patient achieved a MRD-CRi by day 28 after infusion. There was no evidence of blasts in BM either by BM smear or by flow cytometry at serial time points thereafter for 14 months (Fig. 3b and Additional file 1: Figure S1). BM had reconstitution of normal hematopoiesis by day 56 with the exception of platelet count that still unrecovered at a level of 36 × 10^9^/L as the time of this report. Full donor chimerism was established at day 14 post infusion and remained stable thereafter.

### Haplo-TanCAR-T 19/22 cells exhibited substantial expansion and durable persistence in vivo

After infusion, haplo-TanCAR-T 19/22 cells expanded and peaked at a level of 30.7% of circulating T cells at day 12 followed by a contraction phase with a low level of 0.45% of circulating T cells by day 28. This was coincident with the elimination of circulating B cells that were almost undetected at day 28 by flow cytometry. Haplo-TanCAR-T 19/22 cells were still measurable with a low level of 2.29% of circulating T cells and the circulating B cells still had not recovered as the time of this report (Fig. 3c and Additional file 1: Figure S2). Haplo-TanCAR-T 19/22 cells were also present by flow cytometry at all the response evaluation time points in BM obtained at response evaluation, and chronic B cell aplasia was documented (Fig. 3d and Additional file 1: Figure S2). An overall concordance between the expansion and persistence of haplo-TanCAR-T 19/22 cells in PB measured by flow cytometry and qPCR was observed. As the time of this report, TanCAR-19/22 DNA remained detectable on qPCR with 1134 and 396 copies per microgram DNA in PB and BM, respectively (Fig. 3e).

### Toxicity following haplo-TanCAR-T 19/22 cell infusion

#### CRS

**Fig. 4:**
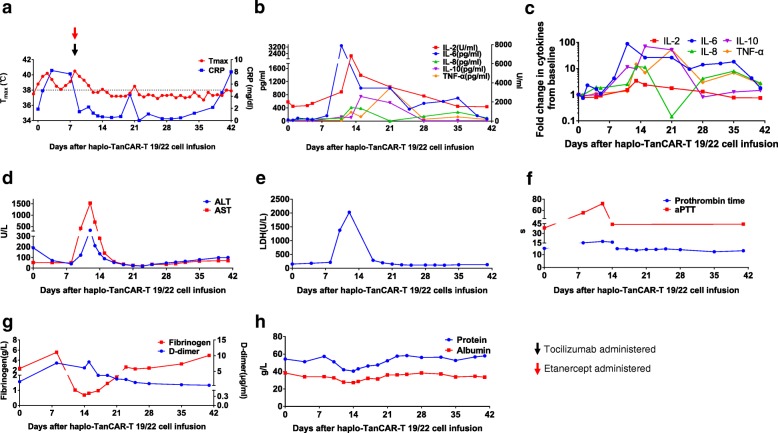
Kinetics of presentation of CRS after haplo-TanCAR-T 19/22 cell infusion. **a** The changes in serum CRP levels and body temperature after haplo-TanCAR-T 19/22 cell infusion. Day 0 is the day of haplo-TanCAR-T 19/22 cell infusion. **b** Concentrations of listed cytokines in serum obtained from patient at the indicated time points. **c** Fold changes of listed serum cytokines from baseline (on day 0 before infusion) after haplo-TanCAR-T 19/22 cell infusion. **d** Serum ALT, AST, **e** LDH, **f** prothrombin time and aPTT, **g** fibrinogen and D-dimer, **h** serum protein and albumin concentrations are shown at the indicated time points after haplo-TanCAR-T 19/22 cell infusion

After haplo-TanCAR-T 19/22 cell infusion, he experienced grade 3 CRS graded according to the UPenn grading scale [21, 22]. Fever of up to 38.8 °C occurred within 24 h after haplo-TanCAR-T 19/22 cell infusion, lasting 11 days and becoming afebrile at day 12 after treated with a lower dose of tocilizumab at 160 mg (1.6 mg/kg) and etanercept 50 mg at day 8 (Fig. 4a). Multiple serum cytokines had markedly increased 7 days post infusion and almost returned to baseline values by day 41 (Fig. 4b, c), where interleukin (IL)-6 levels peaked at 3377 pg/mL (88-fold over baseline) at day 11. Aspartate aminotransferase and lactate dehydrogenase significantly elevated 8 to 10 days after infusion, peaked at 1529.1 U/L (38-fold over upper limit of normal) and 2027.8 U/L (13-fold over baseline) at day 12, respectively, and returned to baseline values by day 21 with best support care (Fig. 4d, e). He also exhibited coagulation dysfunction with prolonged activated partial thromboplastin time, elevated D-dimer, and fallen fibrinogen concentrations, as well as capillary leak with grade 2 hypoalbuminemia in spite of intensive supplementation of protein during the CRS, which resolved by day 23 (Fig. 4f–h).

#### GVHD

**Fig. 5:**
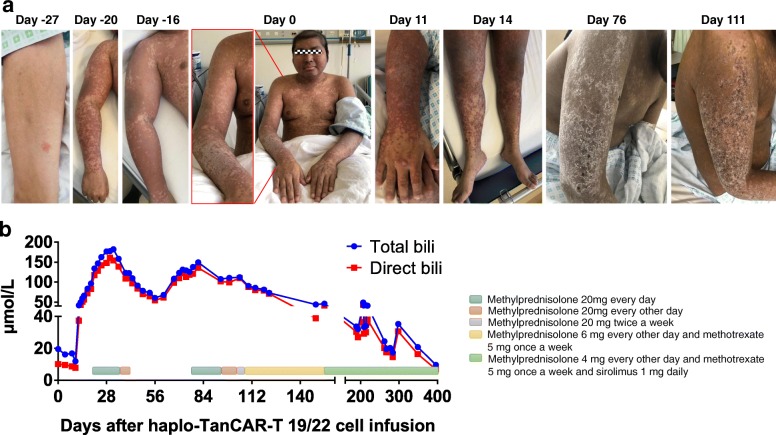
Presentation of GVHD after haplo-TanCAR-T 19/22 cell infusion. **a** Skin GVHD after haplo-TanCAR-T 19/22 cell infusion. The prior stage 3 skin GVHD related to haplo-CAR-T 19 cell infusion worsened after haplo-TanCAR-T 19/22 cell infusion and improved significantly after systemic treatment. Day 0 is the day of haplo-TanCAR-T 19/22 cell infusion. **b** The changes in serum bilirubin levels after haplo-TanCAR-T 19/22 cell infusion, and systemic treatment for GVHD

The prior stage 3 skin acute GVHD that was under control was reactivated and rapidly progressed to stage 4 skin GVHD with new-onset local skin ulcerations particularly in the scrotal skin and mouth mucosa 11 days after haplo-TanCAR-T 19/22 cell infusion (Fig. 5a). The concentration of serum total bilirubin continually elevated from day 12 and increased to 134 μmol/L at day 21 (Fig. 5b). Given the rapidly progressive skin GVHD manifestations and liver involvement, lower-dose methylprednisolone at 20 mg daily as the initial dose with subsequent tapering in an effort to balance the benefits and risks of systemic immunosuppression was implemented from day 21 and discontinued by day 39. Skin rash and serum total bilirubin improved significantly after those treatments. However, stage 3 gut GVHD manifestations mainly including diarrhea occurred from day 50, and the serum total bilirubin elevated again, suggesting a grade 3 acute GVHD. Sixteen doses of methylprednisolone 20 mg per day were administered again from day 78, significantly controlling diarrhea and serum total bilirubin. This patient subsequently exhibited moderate chronic GVHD mainly manifested as scleroderma, diarrhea, and weight loss. Persistent thrombocytopenia with platelet count ranging from 15 × 10^9^ to 43 × 10^9^/L without platelet transfusion could be acknowledged as a manifestation of chronic GVHD in the setting of reconstitution of normal hematopoiesis. The systemic immunosuppressive treatment was tapered within 2 months with methylprednisolone 4 mg every other day and methotrexate 5 mg once a week and sirolimus 1 mg daily as a minimum maintenance dose from day 154 to the time of this report (Fig. 5b), keeping the chronic GVHD under good control.

## Discussion and conclusions

We report an adult patient who had rapidly progressive leukemia after haplo-HSCT with overwhelming disease burden at baseline obtained MRD-CR continuing for more than 14 months with manageable GVHD by taper schedule after treated on haplo-TanCAR-T 19/22 cell protocol. This sustained remission duration could be comparable to that of Novartis’s CTL019 in pivotal ELIANA phase II trial, where the rate of relapse-free survival among 61 patients with a response to treatment was 80% at 6 months and 59% at 12 months, and most of the relapsed patients had CD19-negative disease [23].

Three mechanisms, direct antimalignancy activity of cytoreduction chemotherapy and lymphodepleting chemotherapy, graft-versus-leukemia (GVL) effect mainly mediated by the donor T cells contained in the graft, and targeted immune rejection of leukemia cells with expression of CD19 and/or CD22 by TanCAR-T 19/22 cells, could contribute to the eradication and sustained control of leukemia cells in this case. Among those anti-tumor factors, the key driving force involved in the induction of sustained remission should most possibly be attributed to the TanCAR-T 19/22 cell-mediated specific killing rather than the nonspecific anti-tumor activities raised from GVL effect and/or chemotherapy according to the following characteristics of clinical course of this patient: (1) Haplo-CAR-T 19 cell protocol and haplo-TanCAR-T 19/22 cell protocol were highly similar (as shown in Fig. 1), with the notable exception of in vivo persistence of infused CAR-T cells. Sustained remission was only achieved after infusion of haplo-TanCAR-T 19/22 cells with prolonged persistence rather than haplo-CAR-T 19 cells with transient persistence probably due to unknown abnormal early expansion and steroid use for treatment of sCRS following haplo-CAR-T 19 cell infusion. (2) Prolonged B cell aplasia was observed in this patient. Although the cytoreduction chemotherapy and lymphodepleting chemotherapy also were expected to induce B cell aplasia, but sustained B cell aplasia for more than 14 months with recovery of other blood cell counts seen in this patient only was related to the continued specific immunosurveillance provided by the low level of persisting haplo-TanCAR-T 19/22 cells as shown in Additional file 1: Figure S3. (3) The patient achieved CR by day 14 and MRD-CR by day 28 after haplo-TanCAR-T 19/22 cell infusion, which had been reported in clinical trials of CD19-targeted CAR-T cell for R/R B-ALL [24–26], while the remissions to standard donor lymphocyte infusion, a successful embodiment of GVL effect, were rare with reported CR rates of 0 to 25% [27], and the onset of remission typically occurred over several weeks. Furthermore, the dose of haploidentical CD3+ cells this patient received was one magnitude lower than that of the standard donor lymphocyte infusion dose. Therefore, the initial remission due to the GVL effect for this patient who relapsed after haplo-HSCT was less likely. Collectively, those observations highlighted that TanCAR-T 19/22 cell-mediated specific killing was primarily responsible for the continued remission of this patient. But it must be emphasized that the persistent allogeneic T cell responses suggested by the sustained chronic GVHD could not be ruled out for contributing to the long-term disease control albeit his disease had relapsed after haplo-HSCT in the case of full donor chimerism. Moreover, haplo T cells rather than haplo-TanCAR-T 19/22 cells had advantages in controlling the evolution of CD19- and CD22-double escape variants or clonally related malignancies in other lineages. It will be clearer how TanCAR-T 19/22 cells contribute to the long-term disease control in our well-designed clinical trial of autologous TanCAR-T 19/22 cells in R/R B-ALL.

The main safety concern for this patient after haplo-TanCAR-T 19/22 cell infusion could be the increased risk of the recrudescence of the prior haplo-CAR-T 19 cell infusion-related GVHD that was under control prior to haplo-TanCAR-T 19/22 cell infusion. Not surprisingly, the patient developed grade 3 acute GVHD within 2 months after haplo-TanCAR-T 19/22 cell infusion. How to balance the benefits and risks of systemic immunosuppression was a unique challenge for physician to management of the GVHD this patient experienced. Methylprednisolone at 2 mg/kg/day as the initial dose followed by tapering dose after initial response had been accepted as a standard first-line systemic therapy for acute GVHD [28]. Apparently, this initial dose of methylprednisolone would greatly increase the risk of mediating a greater adverse effect on the anti-tumor activity of haplo-TanCAR-T 19/22 cells; thus, we used a lower-dose methylprednisolone at 20 mg daily as the initial dose followed by elegant titratable dosing in an effort to partially treat GVHD or slow down the GVHD exacerbation but without impair the anti-tumor activity of haplo-TanCAR-T 19/22 cells. The fact indicated that this strategy worked well and reached the effect to be hoped. As observed in this case, steroid exposure would become more frequent and even inevitable in the case of the onset of GVHD; in addition, CRS was always accompanied by acute GVHD in the case of donor-derived CAR-T cells [9], and conditions were difficult to distinguish, making the steroid use more challenging. Here, we established a practical way to titrate GVHD and anti-tumor activity of CAR-T cells, whereby the short-term and long-term clinical response was not affected. It could make sense not only for the management of the GVHD and/or sCRS related to donor-derived CAR-T cells, but also for early immunomodulation for the prevention of severe neurotoxicity.

This application of haplo-TanCAR-T 19/22 cells has provided a demonstration of the potential of inducing durable remission of R/R B-ALL by CAR simultaneously targeting CD19 and CD22, albeit with a clinical experience limited to one case. Moreover, allogeneic CAR therapy in the posttransplant setting potentially confounds the role of TanCAR-T 19/22 cells for this continued remission. Anyway, this finding should encourage continued study of this product, and actually, the well-designed clinical trial of autologous TanCAR-T 19/22 cells in adult patients with R/R B-ALL is now under way. In addition, although second infusion of haplo-CAR-T cells has succeeded in this case, it should be cautioned in other patients particularly in those patients with prior GVHD and must be evaluated case by case.

## Additional file

Additional file 1: **Figure S1.** Bone marrow immunophenotyping at serial time points after haplo-TanCAR-T 19/22 cell infusion. There was no evidence of blasts in BM at day 56 and serial time points thereafter for 14 months. **Figure S2.** Prolonged B cell aplasia after haplo-TanCAR-T 19/22 cell infusion. B cells were eliminated from PB and BM and had not recovered more than 1 year after haplo-TanCAR-T 19/22 cell infusion. Day 0 is the day of haplo-TanCAR-T 19/22 cell infusion. B cells were measured by flow cytometry for CD19 and CD22. **Figure S3.** CD22-specific immunosurveillance mediated by haplo-TanCAR-T 19/22 cells. The circulating CD22+CD19- B cell subclones accounting for 0.56% of circulating lymphocytes at day 95 were undetected by day 105. Day 0 is the day of haplo-TanCAR-T 19/22 cell infusion. B cells were measured by flow cytometry for CD19 and CD22. **Figure S4.** CD19 and CD22 marker expression in BM before haplo-CAR-T 19 cell infusion and haplo-TanCAR-T 19/22 cell infusion. The cells in the D gate represent the blast population count of the total nucleated cells in BM aspirates. (PPTX 3084 kb)

## Data Availability

The datasets supporting the conclusions of this article are included in this published article and its supplementary information files.

## Abbreviations

B-ALL: B cell acute lymphoblastic leukemia
BM: Bone marrow
CAR-T: Chimeric antigen receptor T
CR: Complete remission
CRi: CR with incomplete count recovery
GVHD: Graft versus host disease
GVL: Graft-versus-leukemia
haplo: Haploidentical
HSCT: Hematopoietic stem cell transplantation
IL: Interleukin
MRD: Minimal residual disease
MRD-CR: MRD-negative CR
PB: Peripheral blood
PBMCs: Peripheral blood mononuclear cells
sCRS: Severe CRS
