# Supplementary material for: Haploidentical CD19/CD22 bispecific CAR-T cells induced MRD-negative remission in a patient with relapsed and refractory adult B-ALL after haploidentical hematopoietic stem cell transplantation
Source: J Hematol Oncol. 2019 Jun 10;12:57. doi: 10.1186/s13045-019-0741-6 (PMC6558895; doi:10.1186/s13045-019-0741-6)
Supplement: Supplementary file 1 — Figure S1. Bone marrow immunophenotyping at serial time points after haplo-TanCAR-T 19/22 cell infusion. There was no evidence of blasts in BM at day 56 and serial time points thereafter for 14 months. Figure S2. Prolonged B cell aplasia after haplo-TanCAR-T 19/22 cell infusion. B cells were eliminated from PB and BM and had not recovered more than 1 year after haplo-TanCAR-T 19/22 cell infusion. Day 0 is the day of haplo-TanCAR-T 19/22 cell infusion. B cells were measured by flow cytometry for CD19 and CD22. Figure S3. CD22-specific immunosurveillance mediated by haplo-TanCAR-T 19/22 cells. The circulating CD22+CD19- B cell subclones accounting for 0.56% of circulating lymphocytes at day 95 were undetected by day 105. Day 0 is the day of haplo-TanCAR-T 19/22 cell infusion. B cells were measured by flow cytometry for CD19 and CD22. Figure S4. CD19 and CD22 marker expression in BM before haplo-CAR-T 19 cell infusion and haplo-TanCAR-T 19/22 cell infusion. The cells in the D gate represent the blast population count of the total nucleated cells in BM aspirates. (PPTX 3084 kb) [file 13045_2019_741_MOESM1_ESM.pptx]

## Slide 1
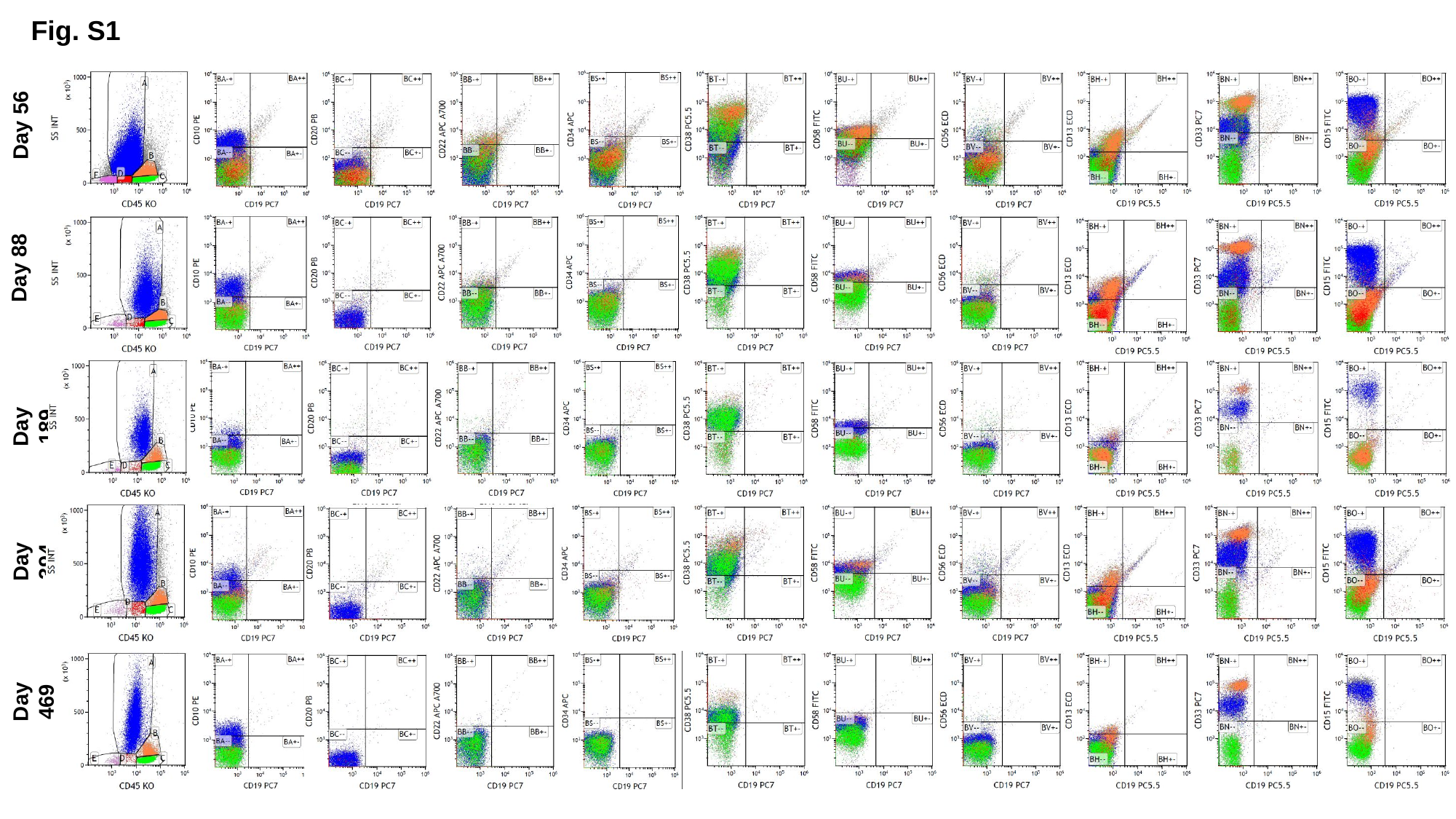

Fig. S1
Day 56
Day 88
Day 189
Day 394
Day 469

## Slide 2
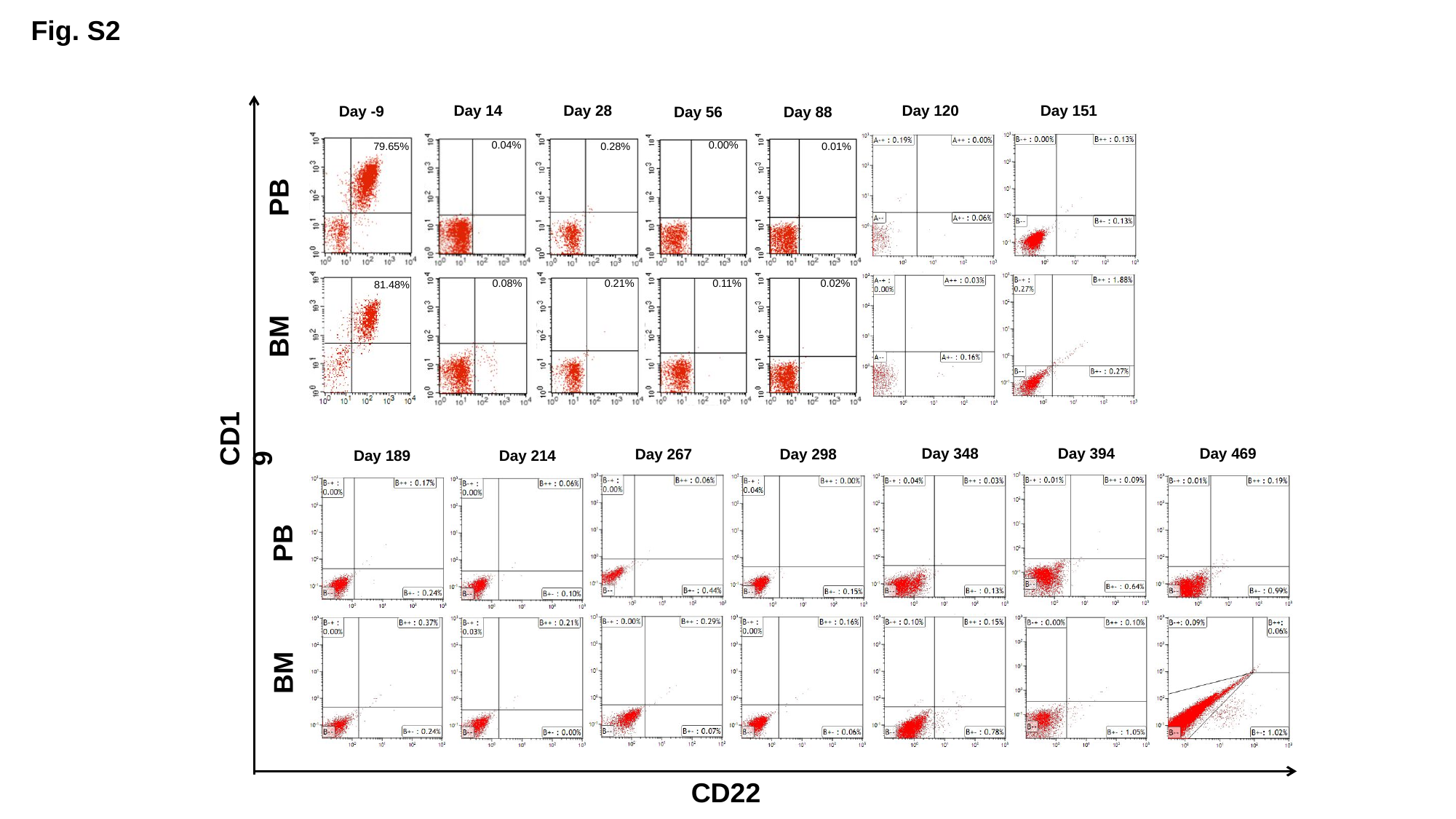

Fig. S2
PB
BM
CD19
BM
CD22
Day 151
Day 14
Day 28
Day 120
Day -9
Day 88
Day 56
Day 348
Day 394
Day 267
Day 298
Day 189
Day 214
PB
0.04%
0.00%
0.01%
0.28%
0.21%
0.11%
0.02%
0.08%
79.65%
81.48%
Day 469

## Slide 3
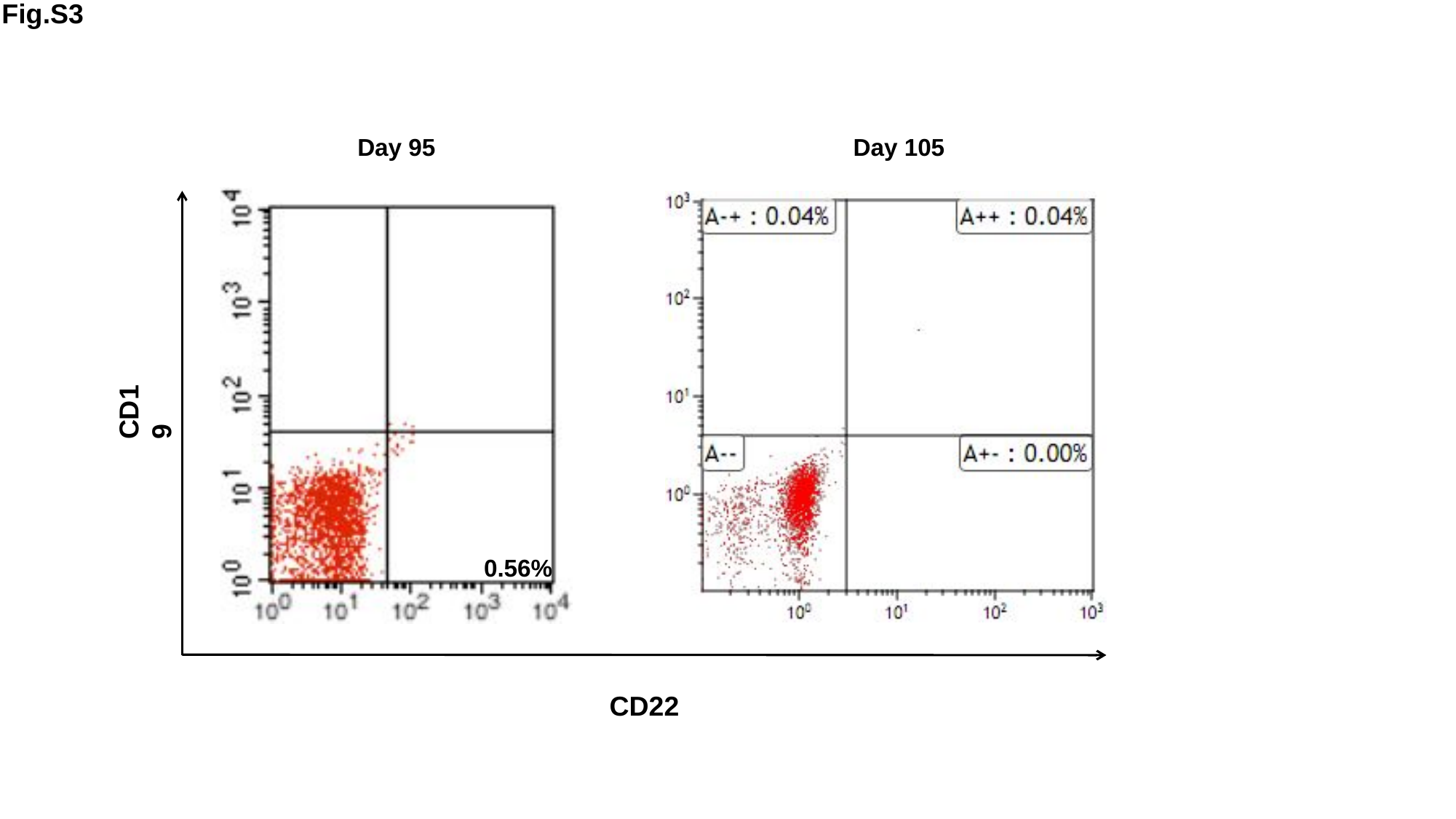

Fig.S3
Day 95
Day 105
0.56%
CD19
CD22

## Slide 4
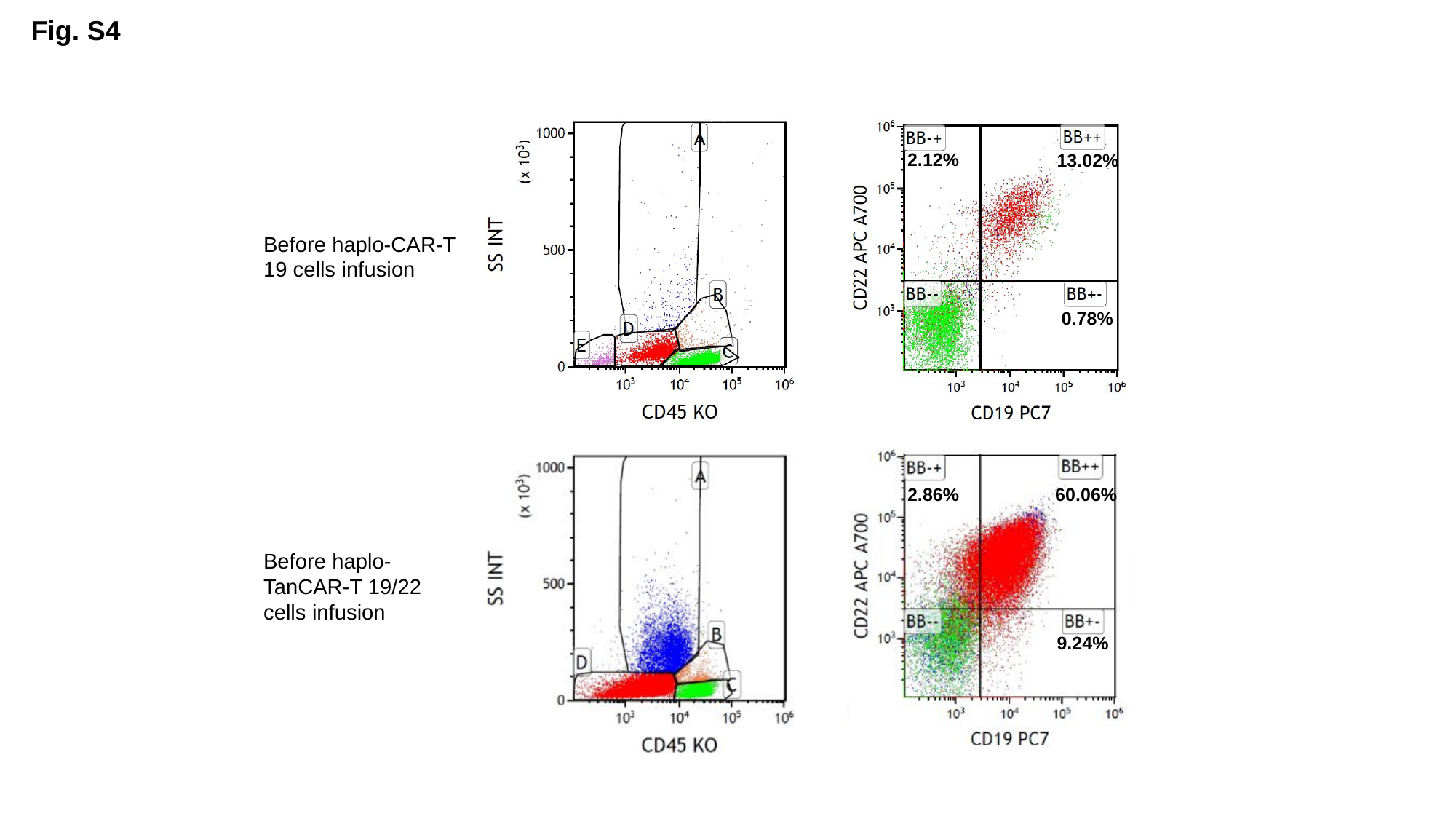

Fig. S4
2.12%
13.02%
Before haplo-CAR-T 19 cells infusion
0.78%
60.06%
2.86%
Before haplo-TanCAR-T 19/22 cells infusion
9.24%
